# Supplementary figures and images for: Diversity in kinetics correlated with structure in nano body-stabilized LacY
Source: PLoS One. 2020 May 7;15(5):e0232846. doi: 10.1371/journal.pone.0232846 (PMC7205474; doi:10.1371/journal.pone.0232846)

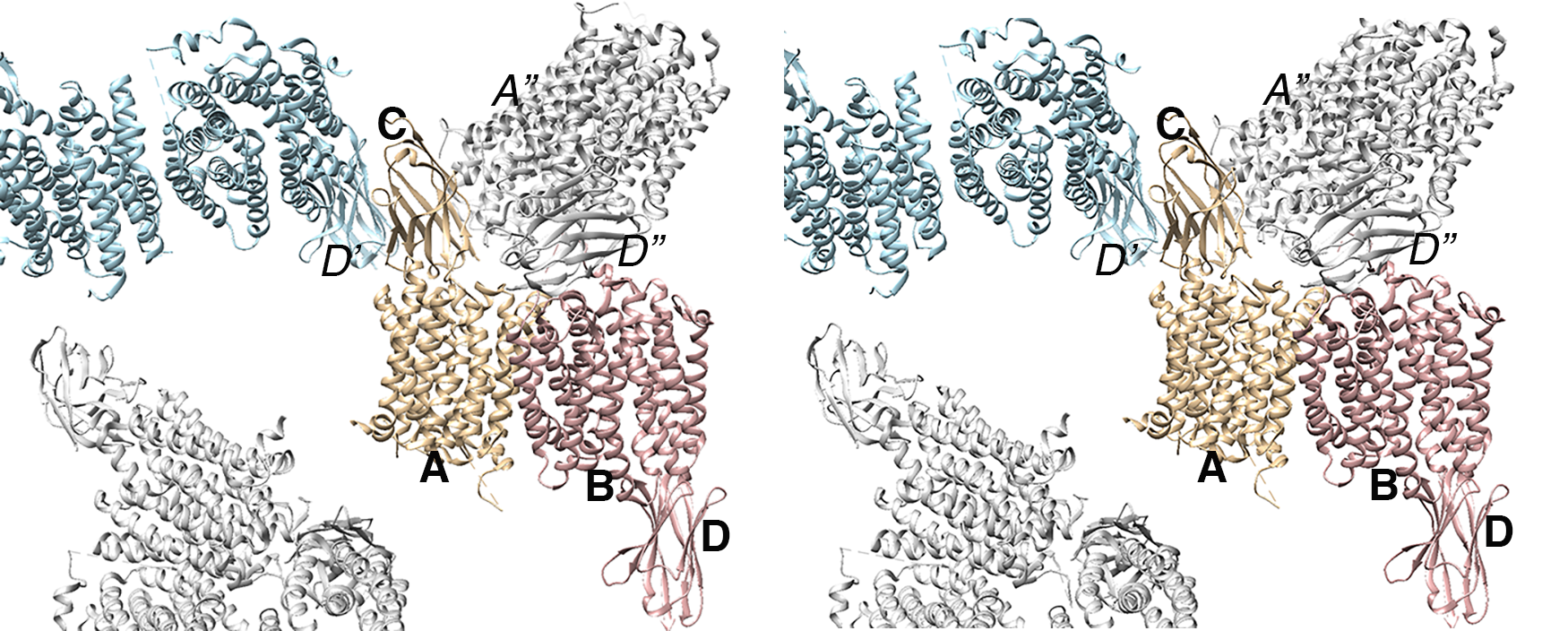

Supplement: S1 Fig — An asymmetric unit of the crystal is shown in crossed-eyes stereo with tan (A-C complex) and brown (B-D complex) ribbons, with chains labeled in bold letters. Two of the symmetry mates in the unit cell that interact with this asymmetric unit are shown as grey and light blue ribbons, with interacting chains labeled with oblique letters. (TIF) [file pone.0232846.s001.tif]

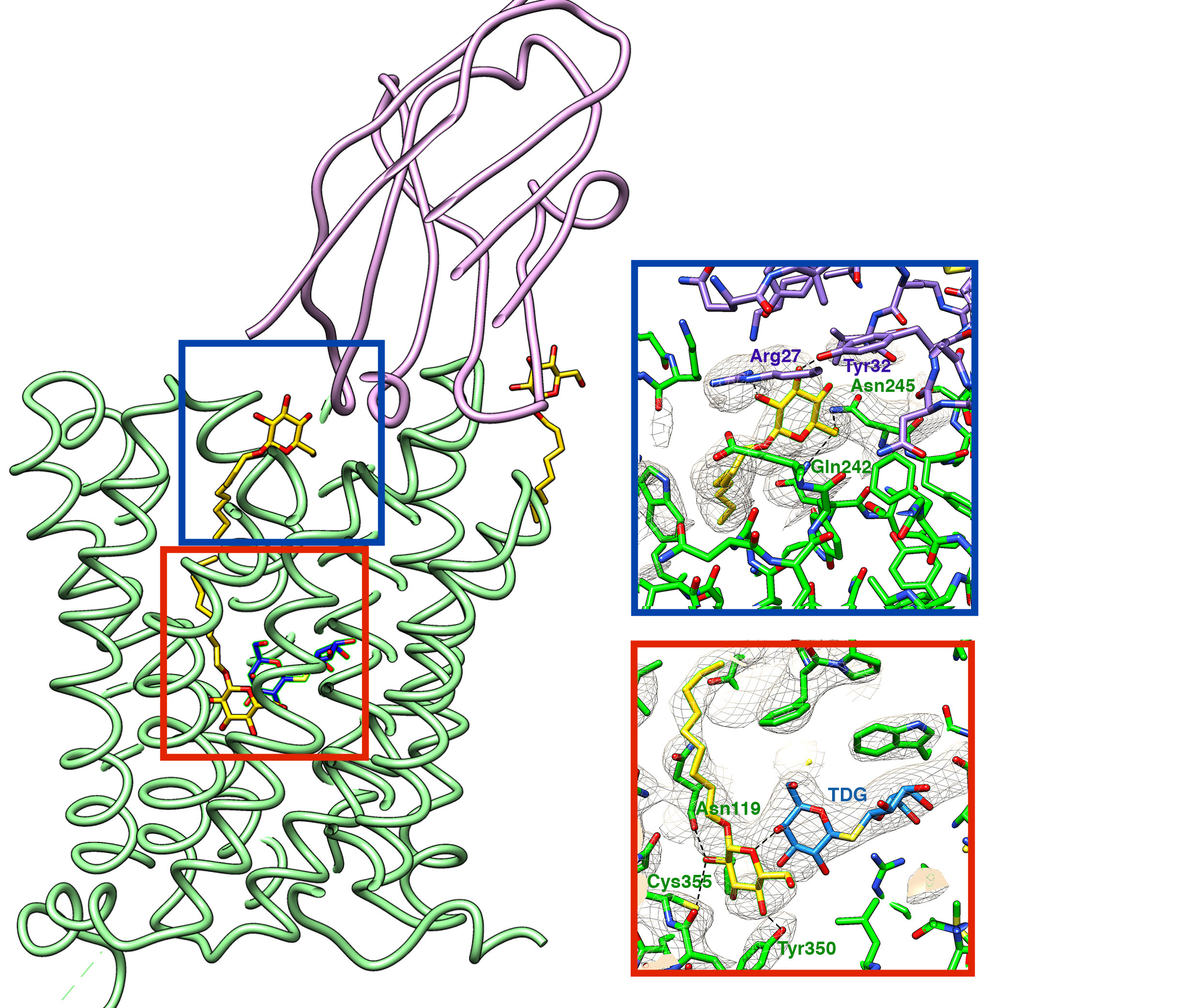

Supplement: S2 Fig — Cartoon version of one LacYWW/TDG/Nb9043 complex (chains A, C) showing β-NG and TDG molecules drawn as sticks and colored by atom type (red oxygens, blue nitrogens, yellow sulfur and gold carbons (for β-NG) or blue carbons (for TDG). LacYWW is colored green and Nb9043 is purple. Two β-NG molecules bound in the periplasmic vestibules, one at the Nb9043 interface and one near the substrate-binding site, are boxed and their interactions with LacYWW are shown on the right, with hydrogen bonds drawn with dashed lines. Regions of a composite omit 2mFo-DFc map surrounding the β-NGs are overlaid with the plots on the right and shown as grey mesh. (TIF) [file pone.0232846.s002.tif]

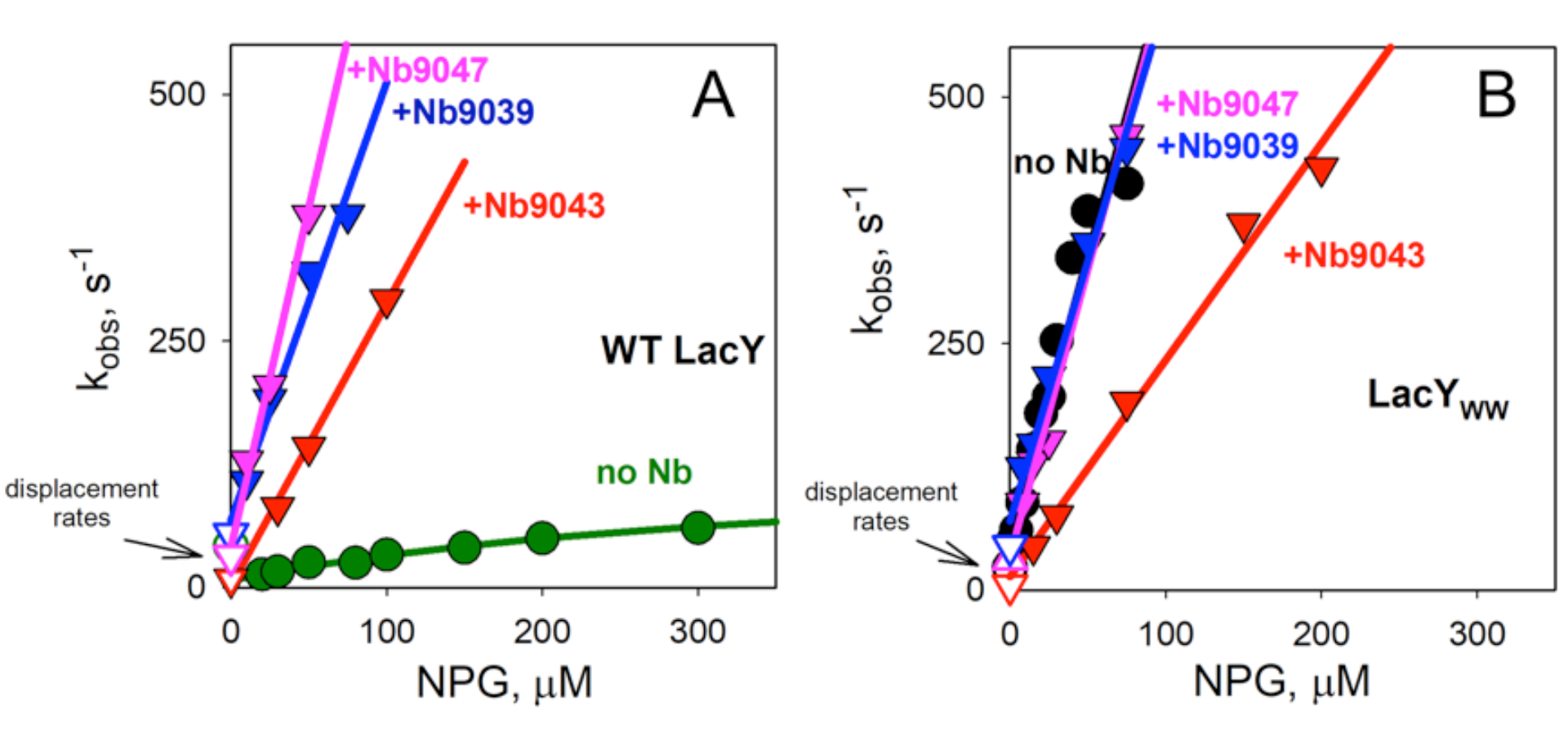

Supplement: S3 Fig — Galactoside binding rates reported by Smirnova et al.[17] were measured by stopped-flow as change in Trp fluorescence utilizing FRET from Trp151 of LacY to bound NPG. Concentration dependencies of the binding rates observed (kobs) were measured before, or after preincubation of LacY with 1.5-fold excess of Nbs. Data shown in blue, pink, and red correspond to LacY complexes with Nb9039, Nb9047, and Nb9043, respectively. Binding rates in the absence of Nb are shown in green and black for WT LacY (A), and LacYww (B), respectively. The kon values were calculated from the slopes of linear fits and presented in S1 Table. (TIF) [file pone.0232846.s003.tif]

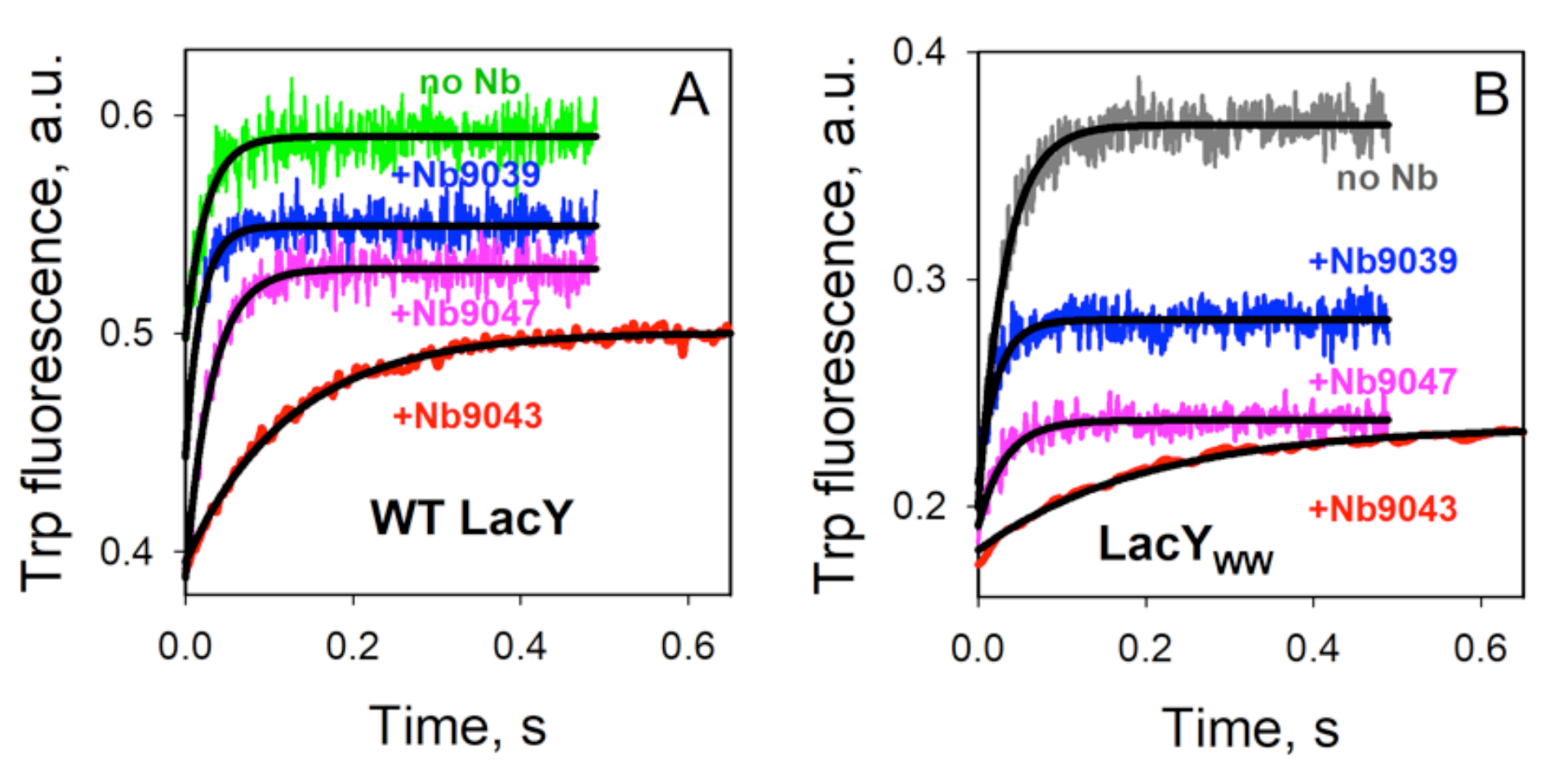

Supplement: S4 Fig — The koff values were measured by stopped-flow as Trp fluorescence increase resulting from displacement of bound NPG (acceptor of FRET from Trp151) by excess of TDG. Single exponential fits (black lines) of stopped-flow traces are shown for WT LacY (A) and LacYww (B) in blue, pink, and red for LacY complexes with Nb9039, Nb9047, and Nb9043, respectively. LacY (0.5 μM) preincubated with 1.5-fold excess of Nbs was mixed first with NPG, and after 10 min with TDG. Final concentrations of NPG and TDG were 0.1 and 10 mM, respectively. The koff values are presented in S1 Table. (TIF) [file pone.0232846.s004.tif]
