# Supplementary material for: Diversity in kinetics correlated with structure in nano body-stabilized LacY
Source: PLoS One. 2020 May 7;15(5):e0232846. doi: 10.1371/journal.pone.0232846 (PMC7205474; doi:10.1371/journal.pone.0232846)
Supplement: S1 Table — (RTF) [file pone.0232846.s005.rtf]

S1Table. Kinetic parameters of galactoside binding to the complexes of LacY, and LacY with Nbs


Nb	WT LacY	LacYww	
	kon
M-1s-1	koff
s-1	kon
M-1s-1	koff
s-1	
no Nb	0.2	41	5.7	31	
Nb9039	4.4	54	5.3	45	
Nb9047	6.9	32	5.8	31	
Nb9043	2.9	8	2.2	5	
