# Supplementary material for: Diversity in kinetics correlated with structure in nano body-stabilized LacY
Source: PLoS One. 2020 May 7;15(5):e0232846. doi: 10.1371/journal.pone.0232846 (PMC7205474; doi:10.1371/journal.pone.0232846)
Supplement: S2 Table — (RTF) [file pone.0232846.s006.rtf]

S2Table.  Data collection and refinement statistics for LacY/TDG/Nb9043
Wavelength	1.115
Resolution range	38.4 - 2.8 (2.9 - 2.8)
Space group	P 65
Unit cell lengths	151.3 151.3 182.5 
Total reflections	165768 (15807)
Unique reflections	57177 (5727)
Multiplicity	2.9 (2.8)
Completeness (%)	96.9 (90.6)
Mean I/sigma(I)	5.8 (0.45)
Wilson B-factor	72
R-merge	0.21 (2.71)
R-meas	0.26 (3.32)
R-pim	0.149 (1.86)
CC1/2	0.987 (0.119)
CC*	0.997 (0.461)
Reflns in refinement	56329 (5233)
Reflns for R-free	1976 (179)
R-work	0.239 (0.379)
R-free	0.275 (0.383)
CC(work)	0.83 (0.29)
CC(free)	0.76 (0.22)
# of non-hydrogen atoms	8425
  macromolecules	8188
  ligands	235
  solvent	2
Protein residues	1031
RMS(bonds)	0.003
RMS(angles)	0.63
Ramachandran favored (%)	96.0
Ramachandran allowed (%)	3.9
Ramachandran outliers (%)	0.1
Rotamer outliers (%)	0.00
Clashscore	5.97
Average B-factor	73.9
  macromolecules	73.3
  ligands	96.3
  solvent	55.2
Statistics for the highest-resolution shell are shown in parentheses.
