# Supplementary material for: Diversity in kinetics correlated with structure in nano body-stabilized LacY
Source: PLoS One. 2020 May 7;15(5):e0232846. doi: 10.1371/journal.pone.0232846 (PMC7205474; doi:10.1371/journal.pone.0232846)
Supplement: Attachment — Submitted filename: Response_to_Reviewers.docx [file pone.0232846.s007.docx]

Dear Dr. van Veen:

We appreciate the reviewers’ careful reading of our submission and have revised the manuscript according to their suggestions, as detailed below.

**Line numbers referred to in this response correspond to the marked-up manuscript.**

**Reviewer #1**

**Line 144, two “Nb9039” Are they Nb9043?**

We changed “Nb9039” to “Nb9043” (line 272)

**Line 217 “antibodies“
The authors described as “antibodies” only once here. For consistency in the terminology, it should be “nanobodies” throughout the paper.**

We changed “antibodies” to “nanobodies” (line 462).

**Line 226, “Nb9047” Is this Nb9039?**

No, it should be Nb9047. We were making the point that although the conformations of the Nb9043 and Nb9047 complexes with LacYww and substrates seen in the crystal structures were nearly identical, in solution without ligands bound they would probably be less similar.

**Line 310, “A degree”
Use the proper font for angstrom.**

This mistake was corrected, line 511.

**Line 315, “sodium citrate 4.5, ” Insert pH as “sodium citrate pH 4.5, ”**

This mistake was corrected, line 515.

**Reviewer #2**

**Major points**
**1) The title is overly complicated, we suggest to simplify it.**

The title was changed to: “Diversity in kinetics correlated with structure in nanobody stabilized LacY”

**2) Line 134 ff: we strongly suggest to analyze the nanobody/LacY interface using the PISA server (or a similar resource). This allows for an objective analysis, including also a calculation of the binding interface, numbers of hydrogen bonds and number of salt bridges. It would be also interesting to see a comparison with the previously characterized nanobodies using the PISA server.**

We analyzed the LacYWW/Nb interfaces of the three structures using PISA and summarize the results starting on lines 273 and 290 of the revised manuscript.

**3) Table S1, Fig. S2 and Fig. S3 show previously published data (Ref 17). It is perfectly fine to include these previously published data as supplementary Figures to have all relevant data complete. However, previous publication of the data needs to be stated clearly in the main text as well as in the respective Figure/Table legends!**

In the revised text (line 493) and in the Figure/Table legends we explicitly state that the data were previously measured by Smirnova et al. and cite reference 17.

**Minor points
1) Line 80/81: Please comment on crystal packing of the two LacY/Nb complexes on the ASU.**

We describe the packing between the two complexes in the ASU and also the crystal contacts between symmetry mates starting at line 197. We also added supplemental figure S1 showing the crystal packing.

**2) Line 84-88: The authors describe in detail, where the two bound β-NG’s are located. It would be nice to see this in a Figure.**

We revised S1 Fig (now S2 Fig) to show the locations of the β-NG’s in the complex as a whole as well as close-ups of the interactions of two β-NG’s in the central cavity with LacYww.

**3) Along the same line: In Figure S1, the authors show the detailed interaction between β-NG and LacYww. However, it would be nice to see a larger picture to know where this interaction takes place and then as a zoom-in one could show what presently is Figure S1. Furthermore, to better distinguish β-NG from LacYww (carbons shown in green for both), the authors could use gold for the β-NG carbons (as in Figure 3d).**

We revised the figure as suggested (see response to minor point 2 above)

**4) Line 83/84: please re-write the sentence “Also poorly ordered, C-terminal residues 412-417 in one molecule of LacYWW and 411-417 in the other were not fitted to density maps.” The sentence is currently difficult to grasp.**

We re-wrote the sentence to simplify it (line 205).

**5) Line 97: remove “LacYWW/TDG/Nb9043 is” before “Differences between LacYww...”**

We corrected this error, line 233.

**6) Line 129-133: Although the second β-NG binding site is not physiological, it would be nice to see this in a figure (maybe in updated Figure S1? See comment above).**

We updated the figure (see response to minor point #2)

**7) Line 144: In Figure 3 you show Nb9043 and not Nb9039.**

We corrected this error, line 272.

**8) Figure 3b: we suggest to label Phe378 and Tyr373.**

We labeled these residues in Figure 3b

**9) Figure 3c: Numbering of the residues from TM7 and TM8 are confusing. It is hard to distinguish whether the numbers belong to the CDR or not (especially: Ala252).**

We have revised Figures 3b-3d so that the colors of the residue labels match the colors of the residues in the plots.

**10) Figure 3d: Not only CDR3 is shown, but also residues from CDR1. In the figure legend, only CDR3 residues are mentioned. But Tyr32 belongs to CDR1, correct? Thr248 is hidden behind Tyr32. It is not clear between which residues the hydrogen bond (dashed line) is. The one between Asn245 and Thr248 or between β-NG and Tyr32?**

We revised Fig. 3d to show Nb9043 side chains only for CDR3. This eliminated the Tyr32 sidechain and allows Thr248 to be seen.

**11) Line 168: It is hard to see the three hydrogen bonds in Fig. 3d (would it be possible to also label Phe251 and Ala252 in Figure 3d?).**

In revised Figure 3d, backbone atoms of the residues that are hydrogen bonded to Arg99 (Thr248, Phe251 and Ala252) are shown as sticks rather than ribbons so that the hydrogen bonds to their carbonyl groups are clearer. We added another hydrogen bond that had been missed in the original submission, from Arg99 NH1 to Phe251O, in both the figure and Table 1. We also labeled Phe251 and Ala252.

**12) Line 188-191: We suggest that the authors show how β-NG binds to the substrate binding site in a Figure.**

The binding mode is shown in revised figure S1 (now S2 Fig).

**13) Line 246: The authors write about an 10-20 fold increased on-rate, whereas in the introduction they wrote that the rate of sugar binding increases by 5-50 fold. Please clarify.**

The 5-50-fold increase refers to a set on 9 nanobodies to LacYww whereas the 10-20-fold increase refers to the set of Nbs discussed in the manuscript. To avoid confusion, we removed “10-20 fold” and only list the on-rates for LacY complexes with these three Nbs and, for comparison, that for uncomplexed LacY (line 495).
